# Pullulan-Coated Iron Oxide Nanoparticles for Blood-Stage Malaria Vaccine Delivery

**DOI:** 10.3390/vaccines8040651

**Published:** 2020-11-03

**Authors:** Liam Powles, Kirsty L. Wilson, Sue D. Xiang, Ross L. Coppel, Charles Ma, Cordelia Selomulya, Magdalena Plebanski

**Affiliations:** 1 Department of Chemical Engineering, Monash University, Clayton, VIC 3800, Australia; liam.powles@gmail.com (L.P.); cordelia.selomulya@unsw.edu.au (C.S.); 2 Department of Immunology and Pathology, Monash University, Melbourne, VIC 3004, Australia; sue.xiang@monash.edu; 3 School of Health and Biomedical Sciences, Royal Melbourne Institute of Technology, Bundoora, VIC 3083, Australia; kirsty.wilson2@rmit.edu.au; 4 Faculty of Medicine, Nursing and Health Sciences, Monash University, Clayton, VIC 3800, Australia; ross.coppel@monash.edu (R.L.C.); charles.ma@monash.edu (C.M.); 5 School of Chemical Engineering, University of New South Wales, Sydney, NSW 2052, Australia

**Keywords:** blood-stage malaria, vaccines, MSP4/5, biodegradable, iron oxide, nanoparticles, CD4+ T cells, antibodies

## Abstract

Vaccines against blood-stage malaria often aim to induce antibodies to neutralize parasite entry into red blood cells, interferon gamma (IFNγ) produced by T helper 1 (Th1) CD4+ T cells or interleukin 4 (IL-4) produced by T helper 2 (Th2) cells to provide B cell help. One vaccine delivery method for suitable putative malaria protein antigens is the use of nanoparticles as vaccine carriers. It has been previously shown that antigen conjugated to inorganic nanoparticles in the viral-particle size range (~40–60 nm) can induce protective antibodies and T cells against malaria antigens in a rodent malaria challenge model. Herein, it is shown that biodegradable pullulan-coated iron oxide nanoparticles (pIONPs) can be synthesized in this same size range. The pIONPs are non-toxic and do not induce conventional pro-inflammatory cytokines in vitro and in vivo. We show that murine blood-stage antigen MSP4/5 from *Plasmodium yoelii* could be chemically conjugated to pIONPs and the use of these conjugates as immunogens led to the induction of both specific antibodies and IFNγ CD4+ T cells reactive to MSP4/5 in mice, comparable to responses to MSP4/5 mixed with classical adjuvants (e.g., CpG or Alum) that preferentially induce Th1 or Th2 cells individually. These results suggest that biodegradable pIONPs warrant further exploration as carriers for developing blood-stage malaria vaccines.

## 1. Introduction

Vaccines against distinct stages of the malaria life cycle have different requirements with respect to the immune responses they need to induce. Vaccine-induced immunity against merozoites (blood-stage malaria parasites) will likely need to mimic naturally acquired immunity, controlling the extent of infection rather than preventing it [1]. Antibodies are the key in such a response, acting to prevent merozoite growth and replication, primarily by inhibiting cell invasion [2]. In addition, recent studies show antibodies can activate the classical complement pathway and opsonize merozoites for phagocytosis or killing by monocytes, macrophages and neutrophils [3,4,5]. Whilst interleukin 4 (IL4) production by T helper 2 (Th2) T cells can support antibody production by B cells, IFNγ production by γδ T cells and T helper 1 (Th1) CD4+ T cells has been suggested to also play a role in mediating blood-stage protection in rodent malaria models [6]. Their importance in human malaria infection remains unclear.

A wide range of blood-stage malaria antigens have been assessed as potential vaccine candidates, with a number of them, notably merozoite surface protein 1 (MSP1) and apical membrane antigen 1 (AMA1), showing limited efficacy in clinical trials [7]. As such, alternative strategies and targets are required. MSP4 and MSP5 are two related *Plasmodium falciparum* glycosylphosphatidylinositol anchored merozoite surface antigens which have seen limited investigation as vaccine targets [2]. MSP4 appears promising for several reasons: firstly, while its function is unknown, it appears to be essential [2]; secondly, in contrast to other surface proteins, it exhibits limited polymorphism [2,8]. Similarly, MSP5 is reported to have no significant polymorphism and naturally acquired antibodies against it have been associated with a reduction in the incidence of clinical malaria [8,9]. Not all findings support the use of MSP4 as in assessments of naturally acquired immunity against arrays of merozoite surface proteins; MSP4 was only weakly associated with protection from symptomatic malaria [10]. It has also been argued that while strongly immunogenic, antibodies against it develop as an early component of naturally acquired immunity; consequently, that makes it a lower-priority vaccine target [11]. The resolution of this argument will require further experimentation. In murine malaria strains, a single homologue of MSP4 and MSP5 with structural similarities, MSP4/5, has been reported [12]. This protein has been shown to protect against challenge with *P. yoelii* when delivered with Freund’s adjuvant and has also been incorporated into DNA vaccine strategies [13,14]. Studies looking at the incorporation of this target with novel delivery systems have been limited. Traditional adjuvants approved for use in humans have proven incapable of inducing immune responses sufficient for use in a malaria vaccine; therefore, other adjuvant/delivery systems are being investigated, such as the use of nanoparticles [15].

Dependent on particle sizes, nanoparticles, such as polystyrene nanoparticles (PSNPs), when used as vaccine carriers, have been shown to be able to enhance humoral responses and induce the production of memory Th1 or Th2 cells against both conjugated protein and peptide [16,17]. PSNPs, in the viral size range of ~40–60 nm, have also shown some protection against murine blood-stage malaria when delivering MSP4/5 and clear infections in an IFN-γ-dependent manner [18]. A unique feature of viral sized PSNPs, compared to other nanoparticle carriers or adjuvant systems, is that they do not induce non-specific inflammatory reactions [17,19,20,21]. This has a number of benefits, amongst them reduction in mild complications from vaccination, such as pain or fever, and an enhanced safety profile for individuals where inducing inflammation can enhance a pre-existing inflammatory disease. It has been shown that the ~40–60-nm PSNPs which best stimulate Th1 responses instead drain to the lymph node and interact with a cross-presenting subset of dendritic cells [17,22]. This contrasts greatly with the many other adjuvants which rely on inflammation to exert their stimulatory effect. If nanoparticle vaccine carriers are to be suitable for human use, then biodegradable alternatives are preferred. Biodegradable non-toxic, non-inflammatory pullulan (an FDA-approved polysaccharide polymer)-coated nanoparticles with magnetic iron-oxide nanoparticle cores (pIONPs) are shown, herein, to be synthesized in the optimal viral size range (40–60 nm) in which PSNPs have the capability to promote cellular immunity. pIONPs were chosen for this study as they are a biodegradable alternative to other biocompatible but non-degradable materials—for example, latex or silica.

Herein, we examine pIONPs’ capability as adjuvant carriers in vaccines against blood-stage malaria by conjugating the pIONPs to the blood-stage antigen MSP4/5. It was observed that the IFN-γ-producing CD4+ memory T cell response was enhanced along with antibody production. This matches the profile required from a blood-stage malaria vaccine, suggesting that pIONPs are a candidate for further investigation towards this application.

## 2. Materials and Methods

### 2.1. Animals

Six- to eight-week-old C57BL/6 or BALB/c mice were purchased from Monash Animal Services, Melbourne, VIC, Australia. All mice were housed in the pathogen-free MICU facility at the Alfred Medical Research and Educational Precinct (AMREP, Melbourne, Australia). The studies presented here were approved by the AMREP Animal Ethics Committee (E1454/2014/M).

### 2.2. Synthesis of Pullulan-Coated Iron Oxide Nanoparticles

pIONPs were synthesized by precipitating ferric chloride and ferrous sulphate in the presence of a large excess of unmodified pullulan. Briefly, 1 g of pullulan was dissolved in 20 mL water and combined with 0.159 g ferric chloride and 0.079 g ferrous sulphate dissolved in 10 mL water. Then, 5 mL of a sodium hydroxide solution created by dissolving 0.2 g sodium hydroxide in 10 mL water was added dropwise over 5 min with stirring. The flask was then covered and heated to 75 °C for an hour. The resulting product was centrifuged to remove large aggregates at 16,200× *g* for 10 min. Carboxymethylation of the pullulan coating was then carried out by dissolving 4 g of sodium hydroxide in the resultant solution with stirring. Following this, 120 mL of isopropanol (Merck, Burlington, MA, USA) was added to this solution, followed by the addition of 2.4 g of sodium chloroacetate over a 5-min period. The solution was heated at 65 °C for 90 min and cooled naturally. The aqueous phase separated from the alcohol and was collected and dialyzed in 100 kDa tubing against saline with at least 4 dialysate changes. The resulting solution was then 0.22-μm filtered to produce the final sterile product.

### 2.3. MTT Toxicity Assay

The Cos-7 cell line used for toxicity assessment is a fibroblast-like line originally derived from monkey kidney cells. For use in assays, cells which were approximately 50–80% confluent were used. Cos-7 cells were cultured in complete RPMI (RPMI supplemented with 10% fetal bovine serum (FBS, Gibco, Waltham, MA, USA), 100 U/mL penicillin/100 µg/mL streptomycin (Gibco), 4 mM L-glutamine (Gibco), 0.02 M HEPES (Gibco) and 0.1 mM 2-mercaptoethanol (2ME, Sigma, St. Louis, MO, USA); all reagents were 0.22-μm sterile filtered prior to addition). For the MTT assay, 20,000 cells/well were plated in 100 μL in a 96-well plate and incubated for 24 h at 37 °C, 5% CO_2_, before treatment. pIONPs were incubated with Cos-7 cells at 37 °C, 5% CO_2_, for 24 h. An amount of 5 μL of a 5 mg/mL MTT (3-(4,5-dimethylthiazol-2-yl)-2,5-diphenyltetrazolium bromide) solution was added per well and the plate was incubated for 4 h at 37 °C, 5% CO_2_. Supernatant was removed and dimethyl sulfoxide added to solubilize the formazan product. The absorbance was measured as that at 690 nm subtracted from that at 570 nm, with viability calculated relative to unstimulated cells.

### 2.4. Bone Marrow Dendritic Cell (BMDC) Culture

C57BL/6 mice were culled by CO_2_ asphyxiation and their femur, tibia and humerus bones were collected, cleaned of tissue and sterilized by soaking in 80% *v/v* ethanol for 1 min. The ends of the bones were removed to expose marrow and the bone was place in a 0.2-mL tube which had been pierced through the base with a 19-gauge needle. This was placed in a microcentrifuge tube and capped. Bone marrow cells were flushed via centrifugation (pulsed to 6000× *g*) and red cells were then lysed with ammonium-citrate-potassium (ACK) buffer. The remaining cells were passed through a 100-μm cell strainer, counted with a hemocytometer and dispersed in complete RPMI supplemented with 10 ng/mL GM-CSF at 5 × 10^5^ cells/mL. Then, 3 mL (1.5 × 10^6^ cells) were plated per well in 6-well plates and incubated at 37 °C, 5% CO_2_ for 3 days before treatment.

### 2.5. Splenocyte Culture

The spleens of C57BL/6 mice were harvested and dissociated through a 100-μm cell strainer with the end of a 3-mL syringe. Red cells were lysed with ACK buffer and leukocytes passed through a 100-μm cell strainer and counted with a coulter counter. For culture, cells were plated at 4 million cells/well in 2 mL complete RPMI in 6-well plates with treatments. For use in enzyme-linked immunospot (ELISpot) assays, cells were dispersed in complete RPMI at 10^7^ cells/mL. For direct staining, 2.5 × 10^6^ cells/well were plated out in 96-well V-bottom plates. Then, 10^6^ pIONPs/cell were incubated with bone marrow dendritic cells (BMDCs) and splenocytes. These were cultured at 37 °C, 5% CO_2_, for 1–24 h.

### 2.6. Flow Cytometry Staining

Cells were resuspended in 30 μL of the Zombie Aqua dye in PBS (Biolegend) in conical ‘V’-bottom plates and stained in the dark for a minimum of 10 min at room temperature. Each well was then washed with 100 μL PBS/2% FBS and centrifuged at 423× *g* for 4 min before the supernatant was discarded. Following staining, cells were fixed in 60 μL PBS/1% paraformaldehyde and analyzed within 72 h. Cells were analyzed with either an LSR II instrument (BD Biosciences, Franklin Lakes, NJ, USA) or a Fortessa X20 instrument (BD Biosciences), both located in the Flow Core facility at AMREP. All analyses were done using Flowlogic (Inivai, Mentone, Australia), provided by the AMREP Flow Core.

### 2.7. Cytokine ELISAs on BMDC Supernatant

IL-6, IL-12p70 and TNFα levels in culture supernatants were assessed using enzyme-linked immunosorbent assay (ELISA) sets from BD Biosciences. IL-1β was assessed using an ELISA set from BioLegend. ELISAs were completed following the manufacturer’s recommendations. Briefly, capture antibodies were diluted as suggested in the correct coating buffer and 100 μL/well were added to Maxisorp ELISA plates (Nunc, Roskilde, Denmark) overnight at 4 °C. Following washing with PBS/0.05% Tween 20, the plates were blocked with 200 µL/well PBS/10% FBS and undiluted supernatant samples and standards (100 μL/well) were added for 2 h. Biotinylated detection antibodies combined with the avidin–horseradish peroxidase (HRP) conjugate were added (100 μL/well) at the suggested dilutions for 1 h. Lastly, 3,3’,5,5’-Tetramethylbenzidine (TMB, Life Technologies, Carlsbad, CA, USA) was added (100 μL/well) to detect HRP for 15–30 min, the reaction was stopped with 1 M HCl (50 μL/well) and the absorbance was measured at 450 nm. For tumour necrosis factor (TNF) and IL1β, the detection step was separated from the avidin step with the solutions added for 1 h and 30 min, respectively, prior to TMB addition.

### 2.8. Antigen Conjugation to Nanoparticles

EDC/NHS chemistry was used to conjugate MSP4/5 (kindly provided by Ross Coppel, Monash University, Australia) to particles. pIONPs, diluted to an estimated 1% solids final concentration, were combined with 4 mg/mL 1-Ethyl-3-(3-dimethylaminopropyl)carbodiimide (EDC) and 25 mM sulfo-NHS and mixed on a rotating wheel for 1 h at room temperature. MSP4/5 was added to a final concentration of 0.95 mg/mL and the solution was mixed for 2 h on a rotating wheel. The reaction was quenched with 7 mg/mL glycine for a minimum of 30 min and dialyzed in 100 kDa tubing (Spectrum Labs, Rancho Dominguez, CA, USA) in 1.8 L, 0.9% saline overnight at 4 °C to remove excess protein. The extent of conjugation was assessed using a microplate Bradford assay (Sigma-Aldrich, St. Louis, MO, USA) following the manufacturer’s recommendations. Purification was performed with an extra 0.22-μm filtration step to ensure sterility.

### 2.9. Immunisations

To assess the capacity of pIONPs to enhance immune responses against MSP4/5, BALB/c mice were vaccinated with different antigen/pIONP/adjuvant combinations twice, two weeks apart. Saline was used for all dilutions as the injection diluent. MSP4/5 was used at an estimated 50 μg/mouse. The adjuvant CpG was used at 25 μg/mouse. Alum was used at 0.3% *w/v*. The formulations were combined and sonicated before intradermal injection (50–110 μL, dependent on formulation) at the base of the tail. Sub-mandibular bleeds were completed one day prior to each injection. Two weeks after the last injection, mice were sacrificed via CO_2_ asphyxiation and cardiac blood and spleens collected for antibody and memory T cell analysis.

### 2.10. Multiplex Assay for Cytokine Analysis

A 21-plex MILLIPLEX assay (Merck) was used to assess cytokine levels in 48-h sera following the manufacturer’s instructions. Briefly, samples were diluted one in two in assay buffer and 25 µL of controls, standards, diluted samples and backgrounds were added to the included 96-well plate with 25 µL of serum matrix (backgrounds, standards and controls) or assay buffer (background and samples). This was combined with 25 µL of the pre-mixed magnetic beads and incubated overnight with agitation on a plate shaker at 4 °C. Supernatant was decanted with beads retained with a magnetic plate and the plate was washed twice with wash buffer. Next, the detection antibodies (25 µL/well) were added for one hour, followed by the streptavidin- phycorerythrin (PE) conjugate (25 µL/well) for 30 min. The plate was washed twice using the magnetic plate and 150 µL/well of sheath fluid was added before the plate was read using a Bio-Plex 200 analyzer (Bio-Rad, Hercules, CA, USA).

### 2.11. IgG ELISA

Following cardiac puncture, blood was left to clot for at least an hour and then serum was separated by centrifugation at 21,100× *g* for 30 min. Anti-MSP4/5 IgG levels were measured using ELISA. The 96-well Maxisorp ELISA plates were coated with 5 μg/mL protein in carbonate buffer (50 μL/well) overnight at 4 °C. They were washed 5–6 times with PBS containing 0.05% Tween (PBS/Tween) and blocked with 5% skim milk powder in PBS (100 μL/well) for 1 h at 37 °C. Following 5–6 washes with PBS/Tween, they were incubated with a range of serum dilutions (50 μL/well) at 37 °C for 1–2 h. IgG was detected with anti-mouse IgG-HRP (50 μL/well, 1:2000 dilution, Zymed, South San Francisco, CA, USA) for 1–2 h at 37 °C, developed with TMB solution (50 μL/well) for 15–30 min and development was stopped with 1 M HCl (50 μL/well). Optical density was then read at 450 nm.

### 2.12. ELISpot

T cell memory responses from immunized splenocytes were quantified using enzyme-linked immunospot (ELISpot) assay. Multiscreen-IP filter plates (MSIPS4510, Merck) were coated with anti-mouse IFNγ antibody (5 μg/mL, 100 μL/well, clone AN18, MABTech, Nacka Strand, Sweden) or anti-mouse IL4 antibody (5 μg/mL, 100 μL/well, BVD4-1D11, BD Biosciences) overnight at 4 °C, washed 5 times with 200 μL/well sterile PBS and blocked with complete RPMI (150 μL/well) for a minimum of 1 h at 37 °C. Then, 15 μg/mL of MSP4/5 was used for recall with 5 × 10^5^ splenocytes. Medium alone was used as the negative control and 1 μg/mL Concavalin A as a positive control. IFNγ was labelled with biotin anti-mouse IFNγ antibody (1 μg/mL, 100 μL/well, R4-6A2-Biotin, MABTech) or biotin anti-mouse IL4 (1 μg/mL, 100 μL/well, BVD6-24G2, BD Biosciences) for 2 h at room temperature. This was then labelled with streptavidin-alkaline phosphatase (ALP) (1:1000, 100 μL/well, 3310-10, MABTech) or ExtrAvidin-ALP (1:3000, 100 μL/well, Sigma-Aldrich) for 1.5 h at room temperature. Spots were developed with an ALP conjugate substrate kit (Bio-Rad, Hercules, CA, USA) and imaged and counted with an ELISpot reader and software (AID, Strasburg, Germany).

### 2.13. CD4+ T Cell Depletion

For MSP4/5 analysis of CD8+ T cells, CD4+ T cells were depleted with CD4+ depletion microbeads (Miltenyi Biotec, Bergisch Gladbach, Germany) following the manufacturer’s recommendations. Briefly, splenocytes from each group were pooled and incubated with the microbeads. The cells were passed through an MS column (Miltenyi Biotech, Bergisch Gladbach, Germany). The cells which were not bound were collected and counted and the efficiency of the process was checked with flow cytometry. The CD4+-depleted splenocytes were then used in the ELISpot analysis, described above.

### 2.14. Statistics

Statistical comparisons were performed using a one-way analysis of variance (ANOVA), t-test or multiple t-tests with corrections for multiple comparisons with GraphPad Prism software (Version 7). Results are expressed as mean ± standard deviation (SD) and replicates and group sizes are indicated in the figure legends.

## 3. Results

### 3.1. Inflammatory Potential and Toxicity of Pullulan Coated Iron Oxide Nanoparticles

**Figure 1:**
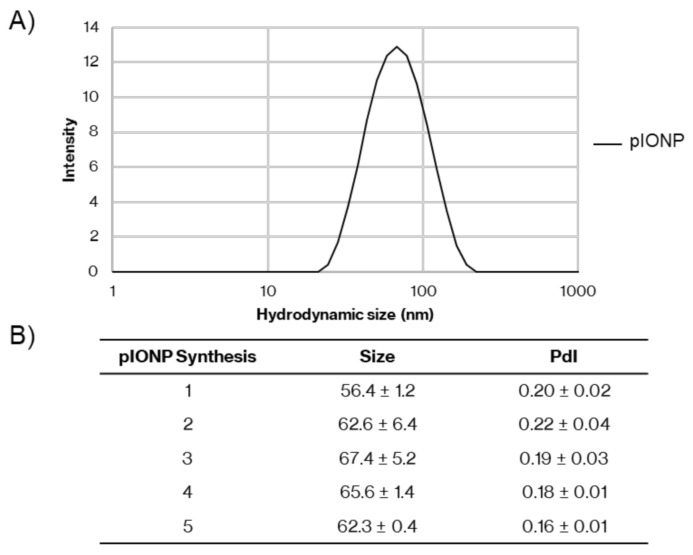
Size of different pullulan-coated iron oxide nanoparticle (pIONP) synthesis batches. Hydrodynamic size of pIONPs. (**A**) Representative plot of hydrodynamic size (intensity-based diameter) of pIONPs in saline, measured by dynamic light scattering (DLS). (**B**) Summary of replicates of particle synthesis with size and polydispersity index (PdI) measured by dynamic light scattering (DLS) in saline. In total, 1–3 independent measurements containing 2–3 cycles of repeated measurements were taken, with SD determined over all replicates.

Polystyrene nanoparticles (PSNPs) offer a powerful self-adjuvanting delivery system for vaccines, enabling the effective stimulation of both humoral and cellular immune responses against the antigen conjugated covalently to the PSNPs [16]. However, being non-degradable has limited their potential effective translation into human vaccines. Herein, pIONPs have been designed to mimic the features of PSNPs that were predicted as key to their effectiveness as self-adjuvanting vaccine carriers. Figure 1 shows that pIONP particles could be made within the size range 56–67 nm (Figure 1) and have a negative zeta potential Z = −27.61.5 mV comparable to PSNPs [16].

**Figure 2:**
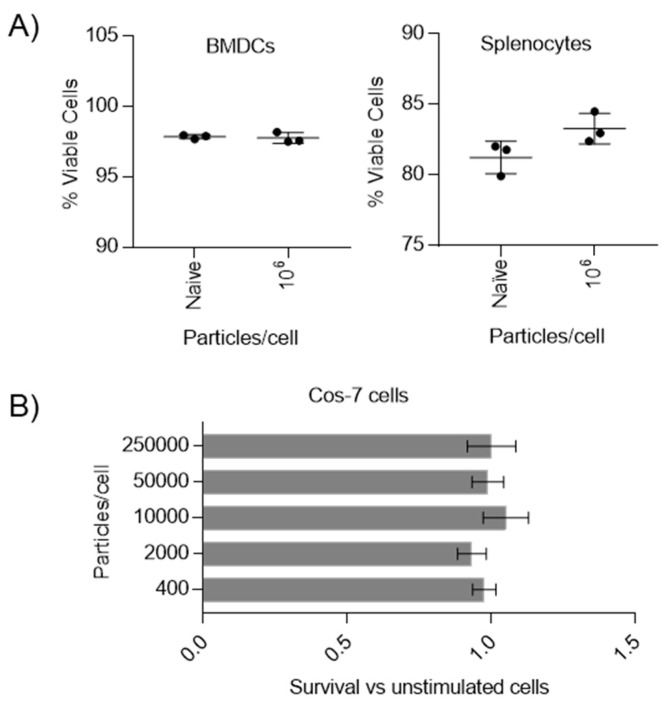
Assessment of pIONP toxicity in various cell types at different concentrations and time points. Nanoparticle toxicity in cultured cells and cell lines. Cell viability was assessed after 4–24 h of incubation using Zombie Aqua and flow cytometry. Data are plotted as mean +/- SD with individual replicates (*n* = 3). (**A**) pIONP toxicity in bone marrow dendritic cells (BMDCs) and splenocytes after 24 and 12 h, respectively. (**B**) pIONP toxicity at a range of concentrations in Cos-7 cells measured by MTT assay and reported as survival relative to cells which were not stimulated with pIONPs. Reported as mean +/- SD, *n* = 4 assay replicates.

To assess their potential for use as vaccine carriers, firstly, the potential toxicity of pIONPs was assessed in vitro across primary cells (including cytokine-derived cell cultures) and immortalized cell lines. Bone marrow-derived dendritic cells (BMDCs) are functionally similar to immature dendritic cells in vivo and are a leading model to study activation and co-stimulation in vitro. Toxicity towards BMDCs was assessed with pIONPs (Figure 2). pIONPs did not decrease cell viability after 24-h incubation with 10^6^ particles per cell, which is more than 10 times the amount of PSNPs of a similar size used previously, and when co-cultured with BMDCs, the PSNPs did not activate signaling pathways [20]. In freshly isolated splenocytes, selected as representative of primary murine immune cells, pIONPs did not decrease and may have increased the percentage of viable cells slightly, albeit not significantly (Figure 2). Finally, cell viability was also assessed via MTT assay in Cos-7 cells, a model widely used in the literature, with pIONPs, again, demonstrating no toxicity at the range of concentrations tested (400—250,000 pIONPs per cell; Figure 2). We conclude that even at high doses, pIONPs are not toxic in vitro and can be assessed further in vivo.

**Figure 3:**
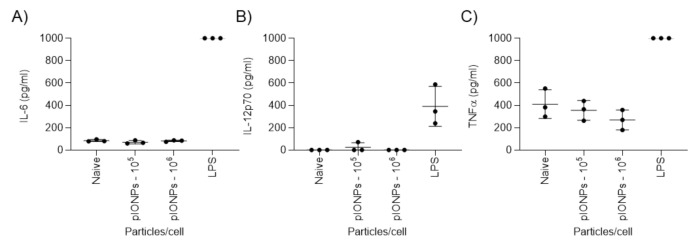
Analysis of cytokine production by BMDCs after 24-h incubation with pIONPs. Inflammatory cytokine levels, (**A**) IL-6, (**B**) IL-12p70 and (**C**) TNF-α, in BMDC supernatants were measured by ELISA. The LPS levels were above the highest standard for IL-6 (**A**) and TNFα (**C**) and were set to 1000 pg/mL. The naïve and 10^6^ pIONPs per cell were below the standard curve for IL12p70 (**B**) and were set to 0 pg/mL.

The presence of foreign material has the capacity to induce an innate immune response under both in vitro and in vivo conditions. This can manifest in changes in cellular surface marker expression or via the induction of cytokines. To determine whether the particles induced the production of inflammatory cytokines, supernatants from BMDC cultures were assessed by ELISA for IL-6, IL-12p70, TNFα and IL-1β. pIONPs did not induce IL-6, IL-12p70 or TNFα production above the level of the unstimulated cells (Figure 3A–C). If anything, TNFα secretion may have decreased slightly when incubated with a high concentration of pIONPs. Furthermore, IL-1β was not detected in any samples bar those stimulated with lipopolysaccharide (LPS) (positive control, data not shown). We conclude that the particles do not induce inflammatory cytokines and do not activate BMDCs in vitro, mimicking PSNPs previously used as carriers.

**Figure 4:**
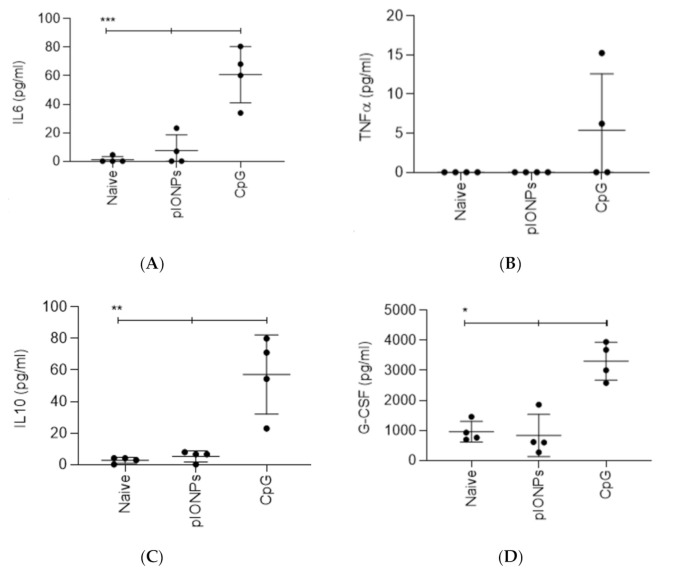

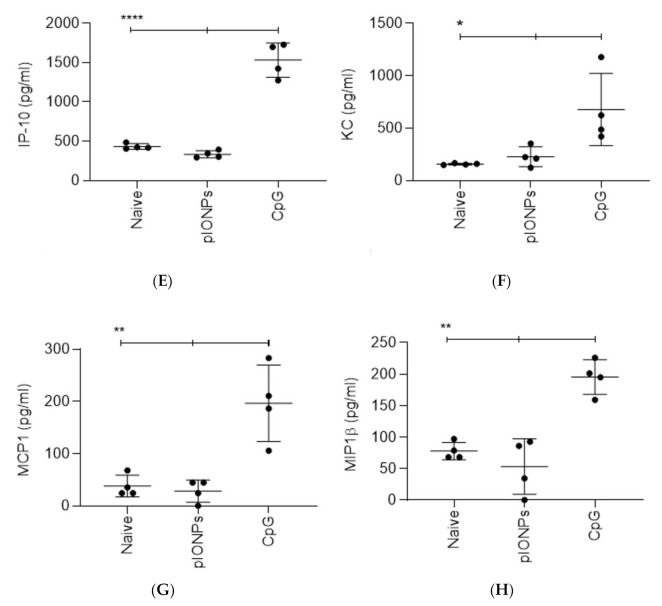
Cytokine responses measured by multiplex assay in sera 48 h after injection of pIONPs. Mice were immunized with pIONPs (1% solids in 100 µL per mouse), CpG (25 µg in 100 µL per mouse) or saline controls (Naïve) intradermally at the base of tail. After 48 h, cardiac serum was harvested and run on a multiplex assay. (**A**) IL-6, (**B**) TNFα, (**C**) IL-10, (**D**) G-CSF, (**E**) IP-10, (**F**) KC, (**G**) MCP-1 and (**H**) MIP-1β. Standards were fit with a five-parameter logistic model and experimental data were interpolated. Individual mice are plotted with mean +/- SD (*n* = 4). All stats were completed using a one-way ANOVA with Tukey’s multiple comparison test. * *p* < 0.05, ** *p* < 0.01, *** *p* < 0.001, **** *p* < 0.0001.

In addition, to test the in vivo potential for an early inflammatory innate response to pIONPs, they were injected in vivo and serum cytokine/chemokine levels were assessed 48 h later by multiplex assay. As predicted from the in vitro results, there were no significant changes in the induction of IL-6, TNFα, IL-10 or G-CSF following immunization with pIONPs (Figure 4). There were also no changes in the chemokines CXCL10/IP-10, CXCL1/KC, CCL2/MCP-1 or CCL4/MIP-1β following pIONP immunization. The CpG positive control induced significant levels above background of G-CSF, IP-10, MCP-1 and MIP1b (Figure 4A–H). These data confirm that pIONPs do not induce an inflammatory cytokine response, neither in vitro nor in vivo, in this rodent species.

### 3.2. Pullulan-Coated Iron Oxide Nanoparticles for Blood-Stage Malaria Antigen Delivery

**Figure 5:**
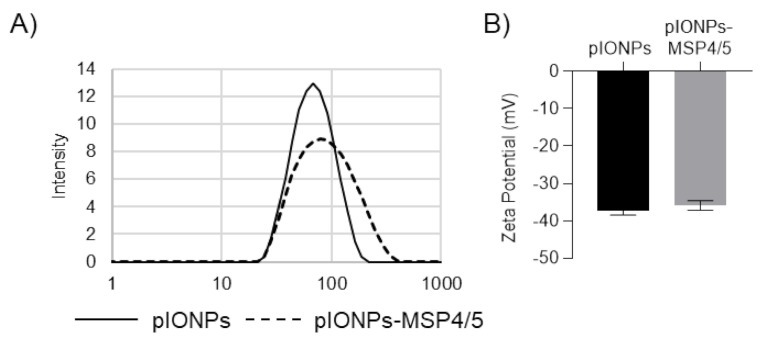
Conjugation of pIONPs to MSP4/5. pIONPs were conjugated to MSP4/5 using EDC chemistry. This led to particles with a final concentration of 83.8 ± 4.2 μg MSP4/5 protein/mg pIONPs. (**A**) Hydrodynamic size of pIONPs before and after conjugation to MSP4/5. (**B**) Zeta potential of the pIONPs before and after conjugation. In total, 1–3 independent measurements were taken, with SD determined over all replicates.

**Figure 6:**
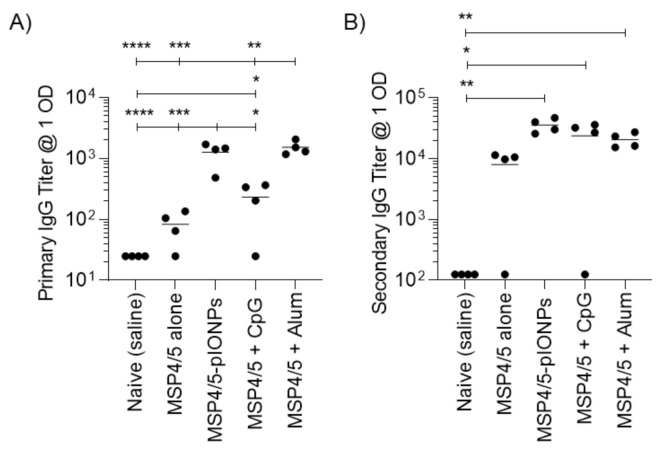
Antibody responses to pIONPs- MSP4/5 compared with the TLR9 agonist, CpG 1826 and Alum. Total IgG responses two weeks after one (**A**) and two (**B**) immunizations (4 weeks total) with MSP4/5 protein (50 µg/per mouse) alone, conjugated to pIONPs (0.57% solids per mouse), mixed with CpG (25 ug per mouse) or mixed with Alum (0.3% final). Titers were calculated by fitting the titration curve with a four-parameter logistic model and interpolating to the selected cut-off, OD = 1. Where the curves did not reach this cut-off, the titer was set at half the lowest dilution. Data presented as individual mice (*n* = 4/group) with mean titer. All stats were completed using a one-way ANOVA with Tukey’s multiple comparison test. ELISA stats were completed on log transformed data to standardize variances. * *p* < 0.05, ** *p* < 0.01, *** *p* < 0.001, **** *p* < 0.0001.

**Figure 7:**
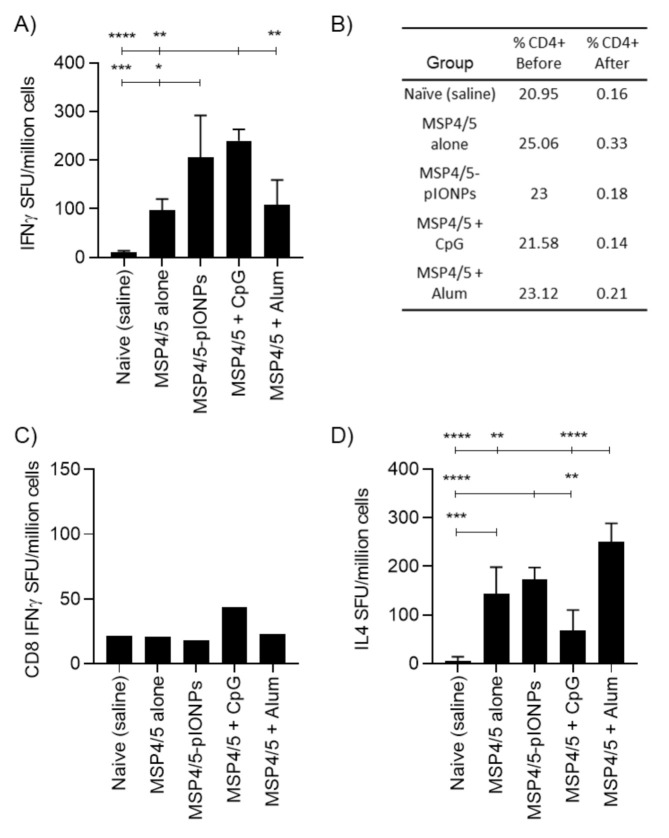
T cell responses to pIONPs—MSP4/5 compared with the TLR9 agonist, CpG 1826 and Alum. (**A**) IFNγ responses measured 2 weeks after the second injection using ELISpot against MSP4/5 protein (50 µg/per mouse), immunized alone, conjugated to pIONPs (0.57% solids per mouse), mixed with CpG (25 ug per mouse) or mixed with Alum (0.3%). Each data point was measured in triplicate and averaged with individual mice presented. Mean +/- SD shown for spot-forming units (SFU)/million cells. (**B**) Percentage of CD4+ cells before and after depletion in each group. (**C**) CD8+-specific IFNγ responses measured using ELISpot against MSP4/5 protein (15 µg/mL) with CD4+-depleted splenocytes. (**D**) IL-4 responses measured using ELISpot against MSP4/5 (15 µg/mL). Mean SFU +/- SD presented from *n* = 4. All stats were completed using a one-way ANOVA with Tukey’s multiple comparison test. * *p* < 0.05, ** *p* < 0.01, *** *p* < 0.001, **** *p* < 0.0001.

Antibody responses are considered vital in combatting blood-stage malarial infection [23]. As such, pIONPs were assessed for their capacity to enhance immune responses against the antigen MSP4/5, derived from *Plasmodium yoelii,* a murine model of malaria infection. pIONPs were covalently conjugated to MSP4/5 using EDC chemistry as described. The size of the particles increased to 79.1 ± 2.5 nm with an increase in polydispersity to 0.25 ± 0.01 (Figure 5). The vaccine adjuvants CpG (TLR9 agonist) and Alum are able to stimulate potent antibody responses. Moreover, CpG as a vaccine adjuvant is known to stimulate high levels of Th1 immunity [24] and Alum is a Th2 immunity-stimulating adjuvant [25]. Therefore, either CpG or Alum were mixed with the MSP4/5 protein as positive controls. Following one injection, pIONPs induced significantly greater anti-MSP4/5 IgG titers than the protein alone or the protein delivered with CpG (Figure 6A). By two immunizations, comparably high anti-MSP4/5 IgG titers, well above background titers, were found, whether immunizing with pIONP-, Alum- or CpG MSP4/5-formulated vaccines (Figure 7A). IFN-γ-producing memory T cell responses (reflective of Th1 immune responses) recalled with the MSP4/5 protein were also assessed. pIONPs induced responses comparable to CpG and significantly greater than the protein alone (*p* < 0.05, Figure 7A). MSP4/5 alone produced T cells at a level significantly greater than the naïve group. To test whether the IFN-γ response was coming from CD8+ T cells or CD4+ T cells, the latter were depleted from the splenocytes before recalling responses with MSP4/5 in the ELISpot assay (Figure 7B). There were no significant responses remaining over background for pIONP-stimulated responses, indicating that the observed total IFN-γ responses had been elicited from Th1 helper T cells and not CD8+ T cells (Figure 7C). The Th2 response was assessed by measuring the elicitation of IL-4-producing memory T cells to MSP4/5 by ELISpot. After two immunizations, pIONPs produced responses comparable to the protein alone and higher than CpG but not Alum (Figure 7D).

## 4. Discussion

Controlling the blood-stage of infection by malaria parasites using vaccines remains a goal within malaria vaccine development. Biodegradable pIONPs in the viral size range have been evaluated for their potential as self-adjuvanting carriers for the murine malaria vaccine antigen MSP4/5. pIONPs were found to be non-toxic in vitro and did not induce the production of inflammatory cytokines, either in vitro or in vivo. Subsequently, the viral-sized pIONPs were further shown to be capable of enhancing both humoral and Th1 responses against the blood-stage malaria antigen MSP4/5, opening the door for potential further exploration as a practical vaccine formulation. Strong, consistent antibody responses comparable to the inflammatory adjuvants CpG 1826 and Alum were present, along with enhanced Th1 responses. The MSP4/5 protein naturally induced moderate Th2 (non-adjuvanted) responses that were decreased by adding CpG, unaffected by pIONPs and, as expected, enhanced by Alum. The end result was a Th1-biased immune response for CpG and Th2-biased for Alum, whereas pIONPs produced a more balanced Th1 and Th2 response whilst still offering comparable induction of Th1 immunity to CpG. The above results make pIONPs a potentially promising carrier for further exploration for the development of malaria blood-stage vaccines.

Future studies would benefit from assessing further the full extent of the different types of immune responses to these particles, including testing for antibody-dependent cellular cytotoxicity (ADCC). Additionally, in the future, other cellular assays, such as investigating Th17 and Tfh cells, would also be interesting to evaluate. In this study, the pIONPs were chemically bound to the MSP4/5 antigen as previous studies in the lab have observed that pIONPs simply mixed with peptide or protein antigens do not induce secretion of IFNγ from T cells (data not shown). Therefore, even in the presence of a soluble peptide, pIONPs alone do not induce IFNγ-mediated immune responses. MSP4/5 is a well-known murine non-toxic blood-stage antigen [13,18,26] and the pIONPs were shown, herein, to be non-toxic at the doses used. Subsequent studies may investigate the difference in response in immunity or toxicity to conjugated versus non-conjugated antigens with pIONPs specifically with MSP4/5 directly as a new research question. However, given the above data and observations, negative controls of pIONPs alone or mixed with MSP4/5 are not likely to yield any significant new data.

Previous studies have examined the immune-stimulating properties of pullulan itself. One study found that pullulan has an adjuvant effect, inducing the upregulation of activation and maturation markers on BMDCs or splenic DCs and inducing the pro-inflammatory cytokines IL-6, IL-12p40 and TNFα [27]. Similarly, another study observed the induction of IFNα, IFNβ1, IL-6 and TNFα mRNA in the plasmocytoid Dendritic Cell (pDC) cell line CAL-1 following incubation of pullulan [28]. The former study used comparable methodology, and in similar pullulan ranges, they described pro-inflammatory cytokine induction, whereas our study does not observe the upregulation of these cytokines. A key difference in their study is the use of free pullulan, compared to our study where pullulan was formed into a biodegradable nanoparticle. Although the specific immune reactive properties of pullulan are beyond the scope of this manuscript, it would be interesting to investigate in future studies.

Biodegradable particles tend to either incorporate antigen into their overall structure, a complicated process, or exceed 100 nm in size [29,30,31,32]. As such, the pIONPs demonstrated here are representative of a promising class of small-sized biodegradable carriers. In other work with non-degradable but biocompatible gold nanoparticles, 30-nm nanoparticles enhanced antibody responses against a conjugated transmission-stage malaria antigen [33]. Similar results have been seen with a range of hydroxyapatite NPs and nanorods [34]. Surface adsorption enabling antigen delivery is key in some cases. For instance, ovalbumin (OVA) adsorption to 70–80-nm nano-dendrimers enhanced the antibody response, mimicking what is seen with covalent conjugation [35].

Typically, adjuvant mechanisms rely on the induction of a pro-inflammatory environment, driving the recruitment of adaptive immune cells and stimulating innate cytokine development, which promotes the activation of adaptive immune responses. Inflammatory adjuvants are often poorly tolerated, inducing excessive systemic inflammation and toxicity in addition to boosting immune responses [36]. In contrast, the pIONPs demonstrated no toxicity or induced inflammatory cytokine production by BMDCs or systemically following in vivo immunization. This indicates a mechanism of immune stimulation distinct from inflammation, likely involving more efficient delivery of antigen to the lymph node and its antigen-presenting cell (APC) populations. Particles less than 100 nm in size have been shown to preferentially drain to the lymph node, whereas larger ones stay at the injection site, relying on migratory and local APC populations for presentation [37].

PSNPs within the 40–100-nm size range are capable of stimulating both humoral and cellular responses to conjugated antigen as well as being non-inflammatory in vitro and in vivo [20,21]. Instead, early immune responses are dominated by granulocyte colony-stimulating factor (G-CSF) and chemokine induction, indicative of an alternative adjuvant mechanism which does not induce conventional pro-inflammatory cytokines (e.g., TNF, IL-6 and IL-1) [38]. By contrast, silica, titanium dioxide, poly(γ-glutamic acid), polyanhydride and carboxymethyl chitosan nanoparticles have all been associated with BMDC activation or pro-inflammatory cytokine secretion [39,40,41,42,43]. The non-inflammatory nature of the particles provides a similar ‘stealth’ backbone to the previously extensively characterized non-degradable PSNP vaccine carriers. As pIONPs are biodegradable, they may offer many additional applications.

## 5. Conclusions

The effective demonstration of pullulan-based nanoparticle carriers capable of enhancing humoral and Th1 immune responses is promising. Their biodegradability and non-toxic, non-inflammatory profiles differ significantly from those of traditional adjuvants. This, combined with their ease of synthesis and their demonstrated ability to stimulate the immune response required against blood-stage malaria, suggests that further investigation of their capabilities in this regard is warranted.

## References

[1] Richards J.S., Stanisic D.I., Fowkes F.J., Tavul L., Dabod E., Thompson J.K., Kumar S., Chitnis C.E., Narum D.L., Michon P. (2010). Association between naturally acquired antibodies to erythrocyte-binding antigens of Plasmodium falciparum and protection from malaria and high-density parasitemia. Clin. Infect. Dis..

[2] Beeson J.G., Drew D.R., Boyle M.J., Feng G., Fowkes F.J., Richards J.S. (2016). Merozoite surface proteins in red blood cell invasion, immunity and vaccines against malaria. FEMS Microbiol. Rev..

[3] Boyle M.J., Reiling L., Feng G., Langer C., Osier F.H., Aspeling-Jones H., Cheng Y.S., Stubbs J., Tetteh K.K., Conway D.J. (2015). Human antibodies fix complement to inhibit Plasmodium falciparum invasion of erythrocytes and are associated with protection against malaria. Immunity.

[4] Stubbs J., Olugbile S., Saidou B., Simpore J., Corradin G., Lanzavecchia A. (2011). Strain-transcending Fc-dependent killing of Plasmodium falciparum by merozoite surface protein 2 allele-specific human antibodies. Infect. Immun..

[5] Joos C., Marrama L., Polson H.E., Corre S., Diatta A.M., Diouf B., Trape J.F., Tall A., Longacre S., Perraut R. (2010). Clinical protection from falciparum malaria correlates with neutrophil respiratory bursts induced by merozoites opsonized with human serum antibodies. PLoS ONE.

[6] Inoue S., Niikura M., Mineo S., Kobayashi F. (2013). Roles of IFN-gamma and gammadelta T Cells in Protective Immunity Against Blood-Stage Malaria. Front. Immunol..

[7] Healer J., Cowman A.F., Kaslow D.C., Birkett A.J. (2017). Vaccines to Accelerate Malaria Elimination and Eventual Eradication. Cold Spring Harb. Perspect. Med..

[8] Polson H.E., Conway D.J., Fandeur T., Mercereau-Puijalon O., Longacre S. (2005). Gene polymorphism of Plasmodium falciparum merozoite surface proteins 4 and 5. Mol. Biochem. Parasitol..

[9] Perraut R., Joos C., Sokhna C., Polson H.E., Trape J.F., Tall A., Marrama L., Mercereau-Puijalon O., Richard V., Longacre S. (2014). Association of antibody responses to the conserved Plasmodium falciparum merozoite surface protein 5 with protection against clinical malaria. PLoS ONE.

[10] Richards J.S., Arumugam T.U., Reiling L., Healer J., Hodder A.N., Fowkes F.J., Cross N., Langer C., Takeo S., Uboldi A.D. (2013). Identification and prioritization of merozoite antigens as targets of protective human immunity to Plasmodium falciparum malaria for vaccine and biomarker development. J. Immunol..

[11] Dent A.E., Nakajima R., Liang L., Baum E., Moormann A.M., Sumba P.O., Vulule J., Babineau D., Randall A., Davies D.H. (2015). Plasmodium falciparum Protein Microarray Antibody Profiles Correlate With Protection From Symptomatic Malaria in Kenya. J. Infect. Dis..

[12] Kedzierski L., Black C.G., Coppel R.L. (2000). Characterization of the merozoite surface protein 4/5 gene of Plasmodium berghei and Plasmodium yoelii. Mol. Biochem. Parasitol..

[13] Goschnick M.W., Black C.G., Kedzierski L., Holder A.A., Coppel R.L. (2004). Merozoite surface protein 4/5 provides protection against lethal challenge with a heterologous malaria parasite strain. Infect. Immun..

[14] Rainczuk A., Smooker P.M., Kedzierski L., Black C.G., Coppel R.L., Spithill T.W. (2003). The protective efficacy of MSP4/5 against lethal Plasmodium chabaudi adami challenge is dependent on the type of DNA vaccine vector and vaccination protocol. Vaccine.

[15] Powles L., Xiang S.D., Selomulya C., Plebanski M. (2015). The Use of Synthetic Carriers in Malaria Vaccine Design. Vaccines.

[16] Mottram P.L., Leong D., Crimeen-Irwin B., Gloster S., Xiang S.D., Meanger J., Ghildyal R., Vardaxis N., Plebanski M. (2007). Type 1 and 2 immunity following vaccination is influenced by nanoparticle size: Formulation of a model vaccine for respiratory syncytial virus. Mol. Pharm..

[17] Fifis T., Gamvrellis A., Crimeen-Irwin B., Pietersz G.A., Li J., Mottram P.L., McKenzie I.F., Plebanski M. (2004). Size-dependent immunogenicity: Therapeutic and protective properties of nano-vaccines against tumors. J. Immunol..

[18] Wilson K.L., Pouniotis D., Hanley J., Xiang S.D., Ma C., Coppel R.L., Plebanski M. (2019). A Synthetic Nanoparticle Based Vaccine Approach Targeting MSP4/5 Is Immunogenic and Induces Moderate Protection Against Murine Blood-Stage Malaria. Front. Immunol..

[19] Wilson K.L., Xiang S.D., Plebanski M. (2015). Montanide, Poly I:C and nanoparticle based vaccines promote differential suppressor and effector cell expansion: A study of induction of CD8 T cells to a minimal Plasmodium berghei epitope. Front. Microbiol..

[20] Karlson Tde L., Kong Y.Y., Hardy C.L., Xiang S.D., Plebanski M. (2013). The signalling imprints of nanoparticle uptake by bone marrow derived dendritic cells. Methods.

[21] Xiang S.D., Wilson K., Day S., Fuchsberger M., Plebanski M. (2013). Methods of effective conjugation of antigens to nanoparticles as non-inflammatory vaccine carriers. Methods.

[22] Gamvrellis A., Gloster S., Jefferies M., Mottram P.L., Smooker P., Plebanski M., Scheerlinck J.P. (2013). Characterisation of local immune responses induced by a novel nano-particle based carrier-adjuvant in sheep. Vet. Immunol. Immunopathol..

[23] Riley E.M., Stewart V.A. (2013). Immune mechanisms in malaria: New insights in vaccine development. Nat. Med..

[24] Chu R.S., Targoni O.S., Krieg A.M., Lehmann P.V., Harding C.V. (1997). CpG oligodeoxynucleotides act as adjuvants that switch on T helper 1 (Th1) immunity. J. Exp. Med..

[25] Kool M., Fierens K., Lambrecht B.N. (2012). Alum adjuvant: Some of the tricks of the oldest adjuvant. J. Med. Microbiol..

[26] Kedzierski L., Black C.G., Coppel R.L. (2000). Immunization with recombinant Plasmodium yoelii merozoite surface protein 4/5 protects mice against lethal challenge. Infect. Immun..

[27] Zhang W., Yu X., Kwak M., Xu L., Zhang L., Yu Q., Jin J.O. (2016). Maturation of dendritic cells by pullulan promotes anti-cancer effect. Oncotarget.

[28] Wang F., Qiao L., Chen L., Zhang C., Wang Y., Wang Y., Liu Y., Zhang N. (2016). The immunomodulatory activities of pullulan and its derivatives in human pDC-like CAL-1 cell line. Int. J. Biol. Macromol..

[29] Oyewumi M.O., Kumar A., Cui Z. (2010). Nano-microparticles as immune adjuvants: Correlating particle sizes and the resultant immune responses. Expert Rev. Vaccines.

[30] Smith D.M., Simon J.K., Baker J.R. (2013). Applications of nanotechnology for immunology. Nat. Rev. Immunol..

[31] Sloat B.R., Sandoval M.A., Hau A.M., He Y., Cui Z. (2010). Strong antibody responses induced by protein antigens conjugated onto the surface of lecithin-based nanoparticles. J. Control. Release.

[32] Gutjahr A., Phelip C., Coolen A.L., Monge C., Boisgard A.S., Paul S., Verrier B. (2016). Biodegradable Polymeric Nanoparticles-Based Vaccine Adjuvants for Lymph Nodes Targeting. Vaccines.

[33] Kumar R., Ray P.C., Datta D., Bansal G.P., Angov E., Kumar N. (2015). Nanovaccines for malaria using Plasmodium falciparum antigen Pfs25 attached gold nanoparticles. Vaccine.

[34] Hayashi M., Aoshi T., Kogai Y., Nomi D., Haseda Y., Kuroda E., Kobiyama K., Ishii K.J. (2016). Optimization of physiological properties of hydroxyapatite as a vaccine adjuvant. Vaccine.

[35] Lu F., Mencia A., Bi L., Taylor A., Yao Y., HogenEsch H. (2015). Dendrimer-like alpha-d-glucan nanoparticles activate dendritic cells and are effective vaccine adjuvants. J. Control. Release.

[36] Wu T.Y., Singh M., Miller A.T., De Gregorio E., Doro F., D’Oro U., Skibinski D.A., Mbow M.L., Bufali S., Herman A.E. (2014). Rational design of small molecules as vaccine adjuvants. Sci. Transl. Med..

[37] Reddy S.T., Rehor A., Schmoekel H.G., Hubbell J.A., Swartz M.A. (2006). In vivo targeting of dendritic cells in lymph nodes with poly(propylene sulfide) nanoparticles. J. Control. Release.

[38] Xiang S.D., Kong Y.Y., Hanley J., Fuchsberger M., Crimeen-Irwin B., Plebanski M. (2015). Nanoparticles modify dendritic cell homeostasis and induce non-specific effects on immunity to malaria. Trans. R Soc. Trop. Med. Hyg..

[39] Kim J.J., Nam J.P., Nah J.W., Jang M.K., Yee S.T. (2014). Immunoadjuvant efficacy of N-carboxymethyl chitosan for vaccination via dendritic cell activation. J. Med. Food.

[40] Kang K., Lim J.S. (2012). Induction of functional changes of dendritic cells by silica nanoparticles. Immune Netw..

[41] Uto T., Akagi T., Yoshinaga K., Toyama M., Akashi M., Baba M. (2011). The induction of innate and adaptive immunity by biodegradable poly(gamma-glutamic acid) nanoparticles via a TLR4 and MyD88 signaling pathway. Biomaterials.

[42] Zhu R., Zhu Y., Zhang M., Xiao Y., Du X., Liu H., Wang S. (2014). The induction of maturation on dendritic cells by TiO2 and Fe(3)O(4)@TiO(2) nanoparticles via NF-kappaB signaling pathway. Mater. Sci. Eng. C Mater. Biol. Appl..

[43] Tamayo I., Irache J.M., Mansilla C., Ochoa-Reparaz J., Lasarte J.J., Gamazo C. (2010). Poly(anhydride) nanoparticles act as active Th1 adjuvants through Toll-like receptor exploitation. Clin. Vaccine Immunol..

